# Supplementary material for: A novel vehicle-mounted sticky trap; an effective sampling tool for savannah tsetse flies Glossina morsitans morsitans Westwood and Glossina morsitans centralis Machado
Source: PLoS Negl Trop Dis. 2021 Jul 19;15(7):e0009620. doi: 10.1371/journal.pntd.0009620 (PMC8321396; doi:10.1371/journal.pntd.0009620)
Supplement: S1 Appendix — (DOCX) [file pntd.0009620.s001.docx]

**Experimental Designs**

***Glossina morsitans morsitans***

| **Table 1. Colour design experiment** | | | | | | | | | | | |
| --- | --- | --- | --- | --- | --- | --- | --- | --- | --- | --- | --- |
|  | Block 1 | Block 2 | Block 3 | Block 4 | Block 5 | Block 6 | Block 7 | Block 8 |  |  | |
| Time | 09-10:00 | 10-11:00 | 11-12:00 | 12-13:00 | 14-15:00 | 15-16:00 | 16-17:00 | 17-18:00 |  | Key | |
| Day 1 | T2 | T3 | T1 | T3 | T1 | T3 | T2 | T1 |  | T1 | All blue |
| Day 2 | T1 | T2 | T3 | T2 | T3 | T2 | T3 | T3 |  | T2 | All Black |
| Day 3 | T3 | T1 | T2 | T1 | T2 | T1 | T1 | T2 |  | T3 | Black-Blue |

| **Table 2. Orientation experiment** | | | | | | | | | | | | |
| --- | --- | --- | --- | --- | --- | --- | --- | --- | --- | --- | --- | --- |
|  | Block 1 | Block 2 | Block 3 | Block 4 | Block 5 | Block 6 | Block 7 | Block 8 | Block 9 |  |  |  |
| Time | 09-10:00 | 10-11:00 | 11-12:00 | 12-13:00 | 13-14:00 | 14-15:00 | 15-16:00 | 16-17:00 | 17-18:00 |  | Key | |
| Day 1 | T1 | T2 | T1 | T2 | T1 | T2 | T1 | T2 | T1 |  | T1 | In line |
| Day 2 | T2 | T1 | T2 | T1 | T2 | T1 | T2 | T1 | T2 |  | T2 | Perpendicular |

| **Table 3: Olfaction experiment** | | |  |  |  |  |  |  |  |  |  |
| --- | --- | --- | --- | --- | --- | --- | --- | --- | --- | --- | --- |
|  | Block 1 | Block 2 | Block 3 | Block 4 | Block 5 | Block 6 | Block 7 | Block 8 |  |  |  |
| Time | 07-08:00 | 08-09:00 | 09-10:00 | 10-11:00 | 11-12:00 | 12-13:00 | 14-15:00 | 15-16:00 |  | Key | |
| Day 1 | T2 | T1 | T2 | T2 | T1 | T1 | T1 | T2 |  | T1 | No attractant |
| Day 2 | T1 | T2 | T1 | T1 | T2 | T2 | T2 | T1 |  | T2 | Butanone + Octenol |

| **Table 4. Vehicle-mounted sticky panel trap vs black-screen fly round experiment** | | | | | | | | | | | |
| --- | --- | --- | --- | --- | --- | --- | --- | --- | --- | --- | --- |
|  |  | Block 1 | Block 2 | Block 3 | Block 4 | Block 5 | Block 6 |  |  |  |  |
| Time |  | 08-09:00 | 09-10:00 | 10-11:00 | 14-15:00 | 15-16:00 | 16-17:00 |  | Key | |  |
| Day 1 |  | T2 | T2 | T1 | T2 | T1 | T2 |  | T1 | BFR |  |
| Day 2 |  | T1 | T1 | T2 | T1 | T2 | T1 |  | T2 | Vehicle-Mounted Sticky Panel Trap |  |

***Glossina morsitans centralis***

| **Table 5. Colour design experiment** | | | | | | | | | | | | | | |
| --- | --- | --- | --- | --- | --- | --- | --- | --- | --- | --- | --- | --- | --- | --- |
|  | Block 1 | Block 2 | Block 3 | Block 4 | Block 5 | Block 6 | Block 7 | Block 8 | Block 9 | Block 10 | Block 11 | Key | |  |
| Time | 07-08:00 | 08-09:00 | 09-10:00 | 10-11:00 | 11-12:00 | 12-13:00 | 13-14:00 | 14-15:00 | 15-16:00 | 16-17:00 | 17-18:00 |  |  |  |
| Day 1 | T2 | T3 | T1 | T3 | T1 | T3 | T2 | T1 | T3 | T2 | T2 | T1 | All blue |  |
| Day 2 | T1 | T2 | T3 | T2 | T3 | T2 | T3 | T3 | T2 | T3 | T3 | T2 | All Black |  |
| Day 3 | T3 | T1 | T2 | T1 | T2 | T1 | T1 | T2 | T1 | T1 | T1 | T3 | Black-Blue |  |

| **Table 6. Orientation experiment** | | | | | | | | | | | | | |
| --- | --- | --- | --- | --- | --- | --- | --- | --- | --- | --- | --- | --- | --- |
|  | Block 1 | Block 2 | Block 3 | Block 4 | Block 5 | Block 6 | Block 7 | Block 8 | Block 9 | Block 10 | Block 11 |  |  |
| Time | 07-08:00 | 08-09:00 | 09-10:00 | 10-11:00 | 11-12:00 | 12-13:00 | 13-14:00 | 14-15:00 | 15-16:00 | 16-17:00 | 17-18:00 | Key | |
| Day 1 | T1 | T1 | T1 | T2 | T2 | T1 | T2 | T1 | T1 | T2 | T1 | T1 | In line |
| Day 2 | T2 | T2 | T2 | T1 | T1 | T2 | T1 | T2 | T2 | T1 | T2 | T2 | Perpendicular |

| **Table 7. Olfaction experiment** | | | | | | | | | | | | | |
| --- | --- | --- | --- | --- | --- | --- | --- | --- | --- | --- | --- | --- | --- |
|  | Block 1 | Block 2 | Block 3 | Block 4 | Block 5 | Block 6 | Block 7 | Block 8 | Block 9 | Block 10 | Block 11 |  |  |
| Time | 07-08:00 | 08-09:00 | 09-10:00 | 10-11:00 | 11-12:00 | 12-13:00 | 13-14:00 | 14-15:00 | 15-16:00 | 16-17:00 | 17-18:00 | Key | |
| Day 1 | T1 | T2 | T2 | T2 | T1 | T1 | T1 | T1 | T1 | T1 | T1 | T1 | No attractant |
| Day 2 | T2 | T1 | T1 | T1 | T2 | T2 | T2 | T2 | T2 | T2 | T2 | T2 | Butanone + Octenol |

| **Table 8. Vehicle-mounted sticky panel trap vs black-screen fly round experiment** | | | | | | | | | | | |
| --- | --- | --- | --- | --- | --- | --- | --- | --- | --- | --- | --- |
|  | Block 1 | Block 2 | Block 3 | Block 4 | Block 5 | Block 6 | Block 7 | Block 8 |  |  |  |
|  | 07-08:00 | 08-09:00 | 09-10:00 | 10-11:00 | 11-12:00 | 12-13:00 | 14-15:00 | 15-16:00 |  | Key | |
| Day 1 | T2 | T1 | T2 | T2 | T1 | T1 | T1 | T2 |  | T1 | BFR |
| Day 2 | T1 | T2 | T1 | T1 | T2 | T2 | T2 | T1 |  | T2 | Vehicle-Mounted Sticky Panel Trap |
