# Supplementary material for: A novel vehicle-mounted sticky trap; an effective sampling tool for savannah tsetse flies Glossina morsitans morsitans Westwood and Glossina morsitans centralis Machado
Source: PLoS Negl Trop Dis. 2021 Jul 19;15(7):e0009620. doi: 10.1371/journal.pntd.0009620 (PMC8321396; doi:10.1371/journal.pntd.0009620)
Supplement: S2 Appendix — (DOCX) [file pntd.0009620.s002.docx]

Raw Data Set

*Glossina morsitans morsitans*

**Table 1. Colour Experiment**

| Day | Block | Treatment | Ambient Temp | Humidity | Female | Male | Count |
| --- | --- | --- | --- | --- | --- | --- | --- |
| D1 | B1 | T2 | 22.3 | 33 | 0 | 5 | 5 |
| D1 | B2 | T3 | 29.1 | 14 | 11 | 34 | 45 |
| D1 | B3 | T1 | 25.4 | 25 | 11 | 37 | 48 |
| D1 | B4 | T3 | 28.9 | 27 | 14 | 86 | 100 |
| D1 | B5 | T1 | 35 | 14 | 3 | 30 | 33 |
| D1 | B6 | T3 | 30 | 23 | 7 | 9 | 16 |
| D1 | B7 | T2 | 30.8 | 22 | 7 | 22 | 29 |
| D1 | B8 | T1 | 29.7 | 22 | 8 | 77 | 85 |
| D2 | B1 | T1 | 19.8 | 48 | 2 | 16 | 18 |
| D2 | B2 | T2 | 25.8 | 35 | 10 | 32 | 42 |
| D2 | B3 | T3 | 29.4 | 27 | 4 | 30 | 34 |
| D2 | B4 | T2 | 30.8 | 18 | 23 | 48 | 71 |
| D2 | B5 | T3 | 30.4 | 17 | 5 | 20 | 25 |
| D2 | B6 | T2 | 36.1 | 12 | 3 | 15 | 18 |
| D2 | B7 | T3 | 28 | 20 | 1 | 24 | 25 |
| D2 | B8 | T3 | 30.3 | 18 | 5 | 45 | 50 |
| D3 | B1 | T3 | 23.5 | 45 | 7 | 21 | 28 |
| D3 | B2 | T1 | 26.1 | 25 | 11 | 37 | 48 |
| D3 | B3 | T2 | 24.9 | 30 | 7 | 29 | 36 |
| D3 | B4 | T1 | 28.7 | 26 | 8 | 31 | 39 |
| D3 | B5 | T2 | 29.5 | 21 | 4 | 15 | 19 |
| D3 | B6 | T1 | 31 | 14 | 1 | 15 | 16 |
| D3 | B7 | T1 | 30.7 | 14 | 3 | 17 | 20 |
| D3 | B8 | T2 | 31 | 12 | 15 | 35 | 50 |

**Table 2. Orientation Experiment**

| Day | Block | Treatment | Panel | Ambient Temp | Humidity | Female | Male | Count |
| --- | --- | --- | --- | --- | --- | --- | --- | --- |
| D1 | B1 | T1 | Left | 20.7 | 39 | 4 | 13 | 17 |
| D1 | B1 | T1 | Right | 20.7 | 39 | 7 | 20 | 27 |
| D1 | B2 | T2 | Front | 24.6 | 25 | 1 | 10 | 11 |
| D1 | B2 | T2 | Back | 24.6 | 25 | 6 | 25 | 31 |
| D1 | B3 | T1 | Left | 33.8 | 10 | 4 | 22 | 26 |
| D1 | B3 | T1 | Right | 33.8 | 10 | 14 | 41 | 55 |
| D1 | B4 | T2 | Front | 28.4 | 17 | 4 | 18 | 22 |
| D1 | B4 | T2 | Back | 28.4 | 17 | 6 | 7 | 13 |
| D1 | B5 | T1 | Left | 24.7 | 26 | 3 | 26 | 29 |
| D1 | B5 | T1 | Right | 24.7 | 26 | 1 | 10 | 11 |
| D1 | B6 | T1 | Left | 23.2 | 33 | 1 | 3 | 4 |
| D1 | B6 | T1 | Right | 23.2 | 33 | 1 | 14 | 15 |
| D1 | B7 | T1 | Left | 21 | 35 | 1 | 11 | 12 |
| D1 | B7 | T1 | Right | 21 | 35 | 3 | 23 | 36 |
| D1 | B8 | T1 | Left | 26 | 29 | 25 | 58 | 83 |
| D1 | B8 | T1 | Right | 26 | 29 | 26 | 49 | 75 |
| D1 | B9 | T2 | Front | 31.6 | 18 | 3 | 8 | 11 |
| D1 | B9 | T2 | Back | 31.6 | 18 | 5 | 1 | 6 |
| D2 | B1 | T2 | Front | 22.1 | 34 | 6 | 27 | 33 |
| D2 | B1 | T2 | Back | 22.1 | 34 | 11 | 8 | 19 |
| D2 | B2 | T1 | Left | 25.5 | 35 | 20 | 82 | 102 |
| D2 | B2 | T1 | Right | 25.5 | 35 | 26 | 58 | 84 |
| D2 | B3 | T2 | Front | 31.5 | 21 | 7 | 51 | 58 |
| D2 | B3 | T2 | Back | 31.5 | 21 | 14 | 6 | 20 |
| D2 | B4 | T1 | Left | 27.4 | 27 | 9 | 31 | 40 |
| D2 | B4 | T1 | Right | 27.4 | 27 | 8 | 25 | 33 |
| D2 | B5 | T2 | Front | 25.1 | 31 | 46 | 137 | 183 |
| D2 | B5 | T2 | Back | 25.1 | 31 | 21 | 51 | 72 |
| D2 | B6 | T2 | Front | 23.4 | 38 | 15 | 65 | 80 |
| D2 | B6 | T2 | Back | 23.4 | 38 | 2 | 13 | 15 |
| D2 | B7 | T2 | Front | 21.1 | 45 | 8 | 33 | 41 |
| D2 | B7 | T2 | Back | 21.1 | 45 | 2 | 20 | 22 |
| D2 | B8 | T2 | Front | 33.5 | 20 | 6 | 38 | 44 |
| D2 | B8 | T2 | Back | 33.5 | 20 | 15 | 45 | 58 |
| D2 | B9 | T1 | Left | 28.6 | 24 | 5 | 8 | 13 |
| D2 | B9 | T1 | Right | 28.6 | 24 | 4 | 13 | 17 |

**Table 3. Olfaction Experiment**

| Day | Block | Treatment | Ambient Temp | Humidity | Female | Male | Count |
| --- | --- | --- | --- | --- | --- | --- | --- |
| D1 | B1 | T2 | 25.9 | 36 | 32 | 95 | 127 |
| D1 | B2 | T1 | 27.2 | 34 | 52 | 100 | 152 |
| D1 | B3 | T2 | 30.3 | 30 | 35 | 82 | 117 |
| D1 | B4 | T2 | 32.5 | 25 | 65 | 159 | 224 |
| D1 | B5 | T1 | 34.2 | 20 | 29 | 47 | 76 |
| D1 | B6 | T1 | 33.5 | 22 | 25 | 35 | 60 |
| D1 | B7 | T1 | 29.3 | 28 | 50 | 60 | 110 |
| D1 | B8 | T2 | 26.5 | 33 | 166 | 340 | 506 |
| D2 | B1 | T1 | 22.8 | 49 | 27 | 78 | 105 |
| D2 | B2 | T2 | 25.2 | 42 | 39 | 91 | 130 |
| D2 | B3 | T1 | 24.5 | 44 | 33 | 86 | 119 |
| D2 | B4 | T1 | 26.1 | 43 | 69 | 172 | 241 |
| D2 | B5 | T2 | 27.5 | 38 | 27 | 78 | 105 |
| D2 | B6 | T2 | 23.7 | 94 | 16 | 26 | 42 |
| D2 | B7 | T2 | 24.4 | 46.5 | 25 | 35 | 60 |
| D2 | B8 | T1 | 21 | 59 | 17 | 30 | 47 |

**Table 4. Vehicle-Mounted Sticky Panel Trap vs Black-Screen Fly Round**

| Day | Block | Treatment | Ambient temp | Humidity | Female | Male | Count |
| --- | --- | --- | --- | --- | --- | --- | --- |
| D1 | B1 | T2 | 21.5 | 62 | 3 | 8 | 11 |
| D1 | B2 | T2 | 24.4 | 51 | 16 | 63 | 79 |
| D1 | B3 | T1 | 33.4 | 31 | 2 | 30 | 32 |
| D1 | B4 | T2 | 27 | 45 | 18 | 57 | 75 |
| D1 | B5 | T1 | 27.9 | 42 | 7 | 34 | 41 |
| D1 | B6 | T2 | 30 | 38 | 25 | 83 | 108 |
| D2 | B1 | T1 | 26.4 | 44 | 1 | 28 | 29 |
| D2 | B2 | T1 | 28.5 | 38 | 3 | 56 | 59 |
| D2 | B3 | T2 | 34.6 | 27 | 60 | 151 | 211 |
| D2 | B4 | T1 | 34.8 | 24 | 6 | 45 | 51 |
| D2 | B5 | T2 | 33 | 28 | 32 | 104 | 136 |
| D2 | B6 | T1 | 32.4 | 29 | 3 | 22 | 25 |

*Glossina morsitans centralis*

**Table 5: Colour Experiment**

| Day | Block | Treatment | Ambient Temp | Humidity | Male | Female | Count |
| --- | --- | --- | --- | --- | --- | --- | --- |
| D1 | B1 | T2 | 27.5 | 34 | 4 | 3 | 7 |
| D1 | B2 | T3 | 25.5 | 35 | 13 | 10 | 23 |
| D1 | B3 | T1 | 33.1 | 24 | 3 | 0 | 3 |
| D1 | B4 | T3 | 39.9 | 14 | 8 | 3 | 11 |
| D1 | B5 | T1 | 38.1 | 16 | 4 | 6 | 10 |
| D1 | B6 | T3 | 35.5 | 16 | 11 | 23 | 34 |
| D1 | B7 | T2 | 36 | 14 | 34 | 14 | 48 |
| D1 | B8 | T1 | 36.1 | 10 | 34 | 8 | 42 |
| D1 | B9 | T3 | 41 | 10 | 31 | 8 | 39 |
| D1 | B10 | T2 | 34.1 | 14 | 96 | 26 | 122 |
| D1 | B11 | T2 | 31 | 19 | 88 | 23 | 111 |
| D2 | B1 | T1 | 28 | 32 | 2 | 3 | 5 |
| D2 | B2 | T2 | 29.2 | 30 | 4 | 5 | 9 |
| D2 | B3 | T3 | 37.6 | 17 | 4 | 7 | 11 |
| D2 | B4 | T2 | 42.1 | 11 | 7 | 3 | 10 |
| D2 | B5 | T3 | 37.4 | 14 | 9 | 7 | 16 |
| D2 | B6 | T2 | 34.6 | 14 | 22 | 7 | 29 |
| D2 | B7 | T3 | 32.2 | 15 | 30 | 17 | 47 |
| D2 | B8 | T3 | 37.7 | 10 | 44 | 10 | 54 |
| D2 | B9 | T2 | 43.2 | 10 | 48 | 15 | 63 |
| D2 | B10 | T3 | 32.6 | 10 | 61 | 18 | 79 |
| D2 | B11 | T3 | 32.3 | 10 | 74 | 25 | 99 |
| D3 | B1 | T3 | 33.1 | 22 | 4 | 5 | 9 |
| D3 | B2 | T1 | 35.5 | 15 | 6 | 7 | 13 |
| D3 | B3 | T2 | 36.3 | 10 | 15 | 7 | 22 |
| D3 | B4 | T1 | 41.2 | 10 | 5 | 4 | 9 |
| D3 | B5 | T2 | 40.9 | 10 | 3 | 2 | 5 |
| D3 | B6 | T1 | 40.7 | 10 | 9 | 4 | 13 |
| D3 | B7 | T1 | 41.6 | 10 | 55 | 23 | 78 |
| D3 | B8 | T2 | 38.4 | 10 | 20 | 7 | 27 |
| D3 | B9 | T1 | 44.3 | 10 | 22 | 3 | 25 |
| D3 | B10 | T1 | 36.1 | 10 | 86 | 25 | 111 |
| D3 | B11 | T1 | 33.4 | 10 | 56 | 19 | 75 |

**Table 6. Orientation Experiment**

| Day | Block | Treatment | Panel | Ambient temp | Humidity | Female | Male | Count |
| --- | --- | --- | --- | --- | --- | --- | --- | --- |
| D1 | B1 | T1 | Left | 33.5 | 13 | 3 | 3 | 6 |
| D1 | B1 | T1 | Right | 33.5 | 10 | 0 | 4 | 4 |
| D1 | B2 | T1 | Left | 41.2 | 10 | 1 | 0 | 1 |
| D1 | B2 | T1 | Right | 41.2 | 10 | 13 | 27 | 40 |
| D1 | B3 | T1 | Left | 46.3 | 10 | 0 | 3 | 3 |
| D1 | B3 | T1 | Right | 46.3 | 10 | 18 | 43 | 61 |
| D1 | B4 | T2 | Front | 44.4 | 10 | 3 | 8 | 11 |
| D1 | B4 | T2 | Back | 44.4 | 10 | 3 | 7 | 10 |
| D1 | B5 | T2 | Front | 44.1 | 10 | 3 | 8 | 11 |
| D1 | B5 | T2 | Back | 44.1 | 10 | 0 | 0 | 0 |
| D1 | B6 | T1 | Left | 47.4 | 10 | 2 | 8 | 10 |
| D1 | B6 | T1 | Right | 47.4 | 10 | 0 | 2 | 2 |
| D1 | B7 | T2 | Front | 41.1 | 10 | 2 | 2 | 4 |
| D1 | B7 | T2 | Back | 41.1 | 10 | 10 | 22 | 32 |
| D1 | B8 | T1 | Left | 46.1 | 10 | 13 | 28 | 41 |
| D1 | B8 | T1 | Right | 46.1 | 10 | 4 | 12 | 16 |
| D1 | B9 | T1 | Left | 49.3 | 10 | 8 | 17 | 25 |
| D1 | B9 | T1 | Right | 49.3 | 10 | 5 | 6 | 11 |
| D1 | B10 | T2 | Front | 38.4 | 10 | 3 | 22 | 25 |
| D1 | B10 | T2 | Back | 38.4 | 10 | 55 | 123 | 178 |
| D1 | B11 | T1 | Left | 36.8 | 10 | 44 | 108 | 152 |
| D1 | B11 | T1 | Right | 36.8 | 10 | 74 | 125 | 199 |
| D2 | B1 | T2 | Front | 31.7 | 22 | 2 | 0 | 2 |
| D2 | B1 | T2 | Back | 31.7 | 10 | 2 | 0 | 2 |
| D2 | B2 | T2 | Front | 40.1 | 10 | 8 | 15 | 23 |
| D2 | B2 | T2 | Back | 40.1 | 10 | 8 | 6 | 14 |
| D2 | B3 | T2 | Front | 40.5 | 10 | 1 | 1 | 2 |
| D2 | B3 | T2 | Back | 40.5 | 10 | 5 | 10 | 15 |
| D2 | B4 | T1 | Left | 46.9 | 10 | 1 | 0 | 1 |
| D2 | B4 | T1 | Right | 46.9 | 10 | 4 | 16 | 20 |
| D2 | B5 | T1 | Left | 44.9 | 10 | 2 | 4 | 6 |
| D2 | B5 | T1 | Right | 44.9 | 10 | 6 | 13 | 19 |
| D2 | B6 | T2 | Front | 45.2 | 10 | 2 | 9 | 11 |
| D2 | B6 | T2 | Back | 45.2 | 10 | 1 | 4 | 5 |
| D2 | B7 | T1 | Left | 37.8 | 10 | 54 | 36 | 90 |
| D2 | B7 | T1 | Right | 37.8 | 10 | 4 | 2 | 6 |
| D2 | B8 | T2 | Front | 42.4 | 10 | 13 | 11 | 24 |
| D2 | B8 | T2 | Back | 42.4 | 10 | 21 | 15 | 36 |
| D2 | B9 | T2 | Front | 40.9 | 10 | 1 | 0 | 1 |
| D2 | B9 | T2 | Back | 40.9 | 10 | 10 | 7 | 17 |
| D2 | B10 | T1 | Left | 40.8 | 10 | 44 | 34 | 78 |
| D2 | B10 | T1 | Right | 40.8 | 10 | 29 | 23 | 52 |
| D2 | B11 | T2 | Front | 35.7 | 10 | 87 | 68 | 155 |
| D2 | B11 | T2 | Back | 35.7 | 10 | 149 | 103 | 252 |

**Table 7. Olfaction Experiment**

| Day | Block | Treatment | Ambient Temp | Humidity | Male | Female | Count |
| --- | --- | --- | --- | --- | --- | --- | --- |
| D1 | B1 | T1 | 27.3 | 38 | 5 | 8 | 13 |
| D1 | B2 | T2 | 32.8 | 29 | 38 | 34 | 72 |
| D1 | B3 | T2 | 41.5 | 18 | 137 | 45 | 182 |
| D1 | B4 | T2 | 39.1 | 20 | 33 | 25 | 58 |
| D1 | B5 | T1 | 42.2 | 15 | 46 | 27 | 73 |
| D1 | B6 | T1 | 42.1 | 15 | 44 | 26 | 70 |
| D1 | B7 | T1 | 41.7 | 15 | 71 | 38 | 109 |
| D1 | B8 | T1 | 40.1 | 14 | 140 | 61 | 201 |
| D1 | B9 | T1 | 44.7 | 10 | 83 | 30 | 113 |
| D1 | B10 | T1 | 38.3 | 16 | 288 | 105 | 393 |
| D1 | B11 | T1 | 34.8 | 20 | 291 | 148 | 439 |
| D2 | B1 | T2 | 31.2 | 32 | 1 | 1 | 2 |
| D2 | B2 | T1 | 32.9 | 25 | 25 | 18 | 43 |
| D2 | B3 | T1 | 35.1 | 22 | 34 | 31 | 65 |
| D2 | B4 | T1 | 41 | 12 | 14 | 3 | 17 |
| D2 | B5 | T2 | 39.2 | 11 | 17 | 12 | 29 |
| D2 | B6 | T2 | 37.5 | 14 | 20 | 19 | 39 |
| D2 | B7 | T2 | 37.3 | 12 | 115 | 68 | 183 |
| D2 | B8 | T2 | 40.7 | 10 | 137 | 71 | 208 |
| D2 | B9 | T2 | 44.7 | 10 | 98 | 45 | 143 |
| D2 | B10 | T2 | 36.5 | 11 | 148 | 67 | 215 |
| D2 | B11 | T2 | 34.2 | 11 | 199 | 105 | 304 |

**Table 8. Vehicle-Mounted Sticky Panel Trap vs Black-Screen Fly Round**

| Day | Block | Treatment | Ambient Temp | Humidity | Male | Female | Count |
| --- | --- | --- | --- | --- | --- | --- | --- |
| D1 | B1 | T2 | 31.1 | 32 | 23 | 11 | 34 |
| D1 | B2 | T2 | 32.7 | 27 | 30 | 9 | 39 |
| D1 | B3 | T1 | 37.7 | 15 | 11 | 2 | 13 |
| D1 | B4 | T2 | 40.4 | 11 | 7 | 10 | 17 |
| D1 | B5 | T1 | 41.5 | 11 | 4 | 3 | 7 |
| D1 | B6 | T1 | 43.7 | 10 | 7 | 3 | 10 |
| D1 | B7 | T1 | 48.4 | 10 | 3 | 1 | 4 |
| D1 | B8 | T2 | 45.5 | 10 | 1 | 4 | 5 |
| D2 | B1 | T1 | 31.8 | 27 | 20 | 10 | 30 |
| D2 | B2 | T1 | 36.3 | 21 | 30 | 6 | 36 |
| D2 | B3 | T2 | 37.1 | 14 | 21 | 14 | 35 |
| D2 | B4 | T1 | 36.5 | 14 | 2 | 3 | 5 |
| D2 | B5 | T2 | 43.2 | 10 | 12 | 20 | 32 |
| D2 | B6 | T2 | 42.1 | 10 | 44 | 21 | 65 |
| D2 | B7 | T2 | 38.1 | 10 | 13 | 6 | 19 |
| D2 | B8 | T1 | 48.9 | 10 | 1 | 0 | 1 |
