# Supplementary material for: A novel vehicle-mounted sticky trap; an effective sampling tool for savannah tsetse flies Glossina morsitans morsitans Westwood and Glossina morsitans centralis Machado
Source: PLoS Negl Trop Dis. 2021 Jul 19;15(7):e0009620. doi: 10.1371/journal.pntd.0009620 (PMC8321396; doi:10.1371/journal.pntd.0009620)
Supplement: Attachment — Submitted filename: Vales comments on PNTD-D-21-00336.docx [file pntd.0009620.s003.docx]

March 14, 2021

**Vale’s comments on PNTD-21-00338 re vehicle-mounted sticky traps**

**Overview and a suggestion**

The MS reports experiments in Zambia to compare: (i) the time-honoured system of sampling tsetse by hand-net catching on a black-screen fly-round and (ii) a few, more novel, systems involving various colours, arrangements and olfactory modifications of a sticky trap mounted on a small truck.

The work is interesting and definitely deserves to be published. That said, however, the investigations are somewhat skimpy, being conducted over a total of just nine days in each of two locations. Had the study been longer it might have been possible to arrange it better and make the investigation more comprehensive, adding much more novelty to the information offered. As matters stand, the outputs of the work are not much more than could have been reasonably predicted. To address properly the business of recommending a standard sort of sampling system, it would be necessary to address the cost, efficacy, convenience, and applicability of more options than the authors consider now.

Maybe the authors are satisfied with no more than that. If, however, they do want to aim for more, perhaps they should use their field data to develop a new way of comparing the efficacy of sampling systems. For example, they might develop an index of cost-effectiveness based on what it costs to catch, say, 1% of the population of tsetse in 1sqkm round about, maybe with eventual caveats for time requirements, the complexity of the systems and the purpose and conditions of sampling. The present MS would not be able to deal with all sampling options. However, it could launch a potentially important concept in the means of identifying and promoting a sampling system to recommend as a standard. The work currently reported by the authors is more about limited comparisons rather than standardisation as such. I will not be in the least offended if the authors reject my suggestion completely.

In any event, to understand more about the availability of flies to the vehicle-mounted sticky trap it would have been necessary to know more about the way that tsetse respond to the vehicle itself, and to compare sticky and electrocuting systems of catching from a vehicle. Some 25 years ago, in the days of the RTTCP, I got a young Zambian lady to perform these very experiments at Kakumbi Research Station in Zambia. I have forgotten the lady’s name, but Alex Maseko will know her. The work she performed involved using electrocuting devices to catch tsetse flying near, and alighting on, various parts of a Land Rover pick-up operated at different speeds and times. I wonder if the data so produced are available, and whether they might help to give more “body” to the present MS. The idea was that she would ultimately use the data to design a malaise trap to fit on a vehicle She never got round to it, so I got the Tanzanians to take up the idea of a malaise trap on a truck. I think it was quite successful and might deserve a mention in the present MS.

**Recommendation**

Accept the MS with minor revision, which should involve consideration of the detailed comments and suggestions offered below.

**Detailed comments and suggestions**

These comments and suggestions were jotted down in the order they occurred while reading through the MS. In places where I lament the failure of the MS to mention a pertinent matter which in fact the authors do cover later, the authors might wish to consider relocating the coverage. It might make for a clearer and calmer read.

The numbers associated with each comment refer to the line numbers on the pdf I was sent by the editorial office.

*Abstract*

26. The BFR might have been the recommended mobile sampling system several decades ago, but very few people might recommend it for savannah tsetse today.

27. If it is used, the BFR commonly allows for catches of tsetse from the ground and men.

30. Should the first mention of Glossina morsitans morsitans be written in full. Short for the next mentioned species is OK.

33. The present MS deals with effectiveness not efficiency. Efficiency is best employed to refer to the catch as a proportion of what is available to be caught.

36-36. If the term “catch index(ices)” is to be used, should the reference catch be made clearer. Thus, the reader might get the first impression that the reference is the BFR, but then it looks like maybe the reference is one of the screens. It gets especially confusing later when “index” gets mixed up with “times higher”.

36 (Here and throughout). Would “transverse” be better than “perpendicular”. It could be considered that all screens were “perpendicular” in the sense of being upright.

38. No significant effect.

41. Comma respectively.

43 Delete “We conclude that” .

44. is several times more effective for.

45. Their = its.

*Author Summary*

51 to 55. The standardization aspect is not very clear. For example, you imply eventually that any of the colours tested will do, which tends to imply that standardization within the variables you tested is of little consequence. Perhaps the most important thing(s) to standardise are the size/shape/colour/speed of the vehicle – a matter to which you seem to have given no attention whatsoever. In particular, the vast majority of the surface of the mobile bait you used looks to have been the silverish truck. How does silver compete with blue or black Would the relative effectiveness of the screen colours change if the truck was, say, dull black or bright blue?

*Introduction*

72. Strictly speaking the genus Trypanosoma is not the causative agent. Instead it is a few of the species within that large genus.

75. 30, 000 per year in 19?? 000 to >1000 in 20??.

76. AAT is a significant cause.

77. estimated to cause three.

84. G. m. morsitans.

86, You seem not to have indicated that nagana = AAT.

92. Cost-effective tools for sampling tsetse are …… Bear in mind that vehicles and fuel are pricey .

96. “stimuli” not “responses”.

97 Delete “primarily”?

99. the most attractive from a distance, whereas…. Delete comma at end of line.

100-101. The attractiveness of shapes increases in the order …….

102. Is “consequently” right here. If certain settings for various features are shown to result in greatest catches, the fact that traps with a range of settings are used is more of a surprise than a consequence.

107. Delete “Volatile”. All olfactory cues are volatile. Remove the split infinitive. Quantify the range of what is meant by “dramatic”.

109. Delete “other”.

111. their relatively low cost. If you are worried about cost, why not say that cow urine can replace the expensive phenols?

116. Traps give a good idea of daily activity patterns because flies must be very active to visit the traps. Against this, mobile devices go the flies, which then need to do little more than flop off their resting site to arrive at passing baits. A single trap might catch less than a vehicle mounted trap per unit time, but many traps can be bought for the cost/hire of a truck, and traps can be left in place for several days or weeks before needing human attention. Dr William Shereni, in Zimbabwe, arranges his traps for inspection at intervals of up to 3- months. How many vehicle-hours or miles would be needed to catch as many flies as, say, a set of a dozen odour-baited traps operated for a month or so?

118. This is a meaningless statistic as stated here. Does it refer to $283 per 1 trap per 1sqkm per day – or what? For sampling, one worries somewhat more about the cost per sampled fly of the sex/species/age/physiological state that is pertinent.

119. The use of traps can hardly be “prohibitive” since it has long been the standard system operated by a severely cash-strapped Zimbabwe.

119-120. Has the sampling of tsetse really been geographically restricted? I suspect that tsetse have been sampled over areas greater than those sampled for most, even any, mosquito species/subspecies. I suspect also that the cost of genetic analysis of a tsetse might be effectively several times more pricey than actually getting the fly. Are geneticists or other students of fly biology worried about getting flies that are “stuck up”?

122. Use “translocation”. “Movement” can mean no more than a little flapping/wobbling of a screen, which is known not to be particularly attractive. Remove the split infinitive.

123-124. In general, a mobile bait is more effective than a stationary bait for catching G. m. However, it depends on what sort of mobile bait you are using, what sort of stationary one, and the condition of the fly you are after.

125. vehicle-mounted electric taps …..

126. The VET cannot be desperately impractical if it has been used widely and long for sampling in impoverished Zimbabwe.

131. That is indeed true, provided the mobile device you develop is indeed more COST-EFFECTIVE than any other mobile or stationary device, and provided you demonstrate its superiority in that regard.

135. The BFR might be standard in Zambia, but it is widely believed to be somewhat backward for savannah tsetse.

*Materials and methods*

144. Delete “located”.

145. Does the reader really care much about the climatic details. Indeed, will the reader pounce with much eagerness on Fig1 and the details of the Zambian distribution? Are these things just “packing”?

156. Conducting the study over a mere nine days is a bit thin. Presumably, July was sunny and cold. The other work was conducted for nine days in September. It would have been sunny but warmer then. Is there any reason to suspect seasonal effects -- perhaps when clouds alter the illumination of the screens? Was there a difference in catch between the predominantly sunny and predominantly shady sides of the screen?

170. You will get in the bad books of Vestergaard Frandsen if you do not spell the name correctly. Did you rally deal with the Danish, as against the Swiss, arm of the company?

171. As stated here, the screen was covered with cloth on one side, but had no cloth on the other. Is that really right?

172. “strip” not “stripe”? I did not see mention of how long the sticky film was used before replacement. How often must it be replaced under various operating conditions?

182. I am nervous of this reflectance business since there is the plain fact shown, by field experiments, that tsetse are reluctant to alight on shiny surfaces -- and sticky surfaces are shiny. How do the authors talk their way around this? The reactions of a tsetse are more directly pertinent to the present case than the outputs of a spectrometer or any other machine.

198. The sentence here is too long and complex, and is not helped by the string of confusing adjectives/nouns in “best panel trap colour design”.

202. outlineD. It is not clear what order has been outlined above. It is unclear what is meant by the results of one experiment feeding into the next.

203 (here and elsewhere). k not K, in km.

203 to 236. Many of the descriptions given here tend to be very confusing. For example, is it the truck or the BFR that went along the transects? It seems from Fig 2 that the transects were much longer than 300m. Much time was spent waiting, and the VST catching was performed only over 300m. Hence, how much of the catch was represented by flies that visited the stationary truck and got caught when the vehicle moved off? Is that really the way that a VST would be operated in normal surveys? The placement of odour attractants described might have been used by Willemse but I am not sure that it should be regarded as “standard”.

214. “Vehicle fly round” is a term new to this MS. Does it mean a VST.

222. “Speciate” means to evolve into a distinct species.

231 and 236. Why use “tsetse” in some places and “tsetse fly” in others. The former is preferable, being more concise.

238. The stats section is skimpy. I would prefer more details of what was actually done by the packages. For example, were the catches transformed for analysis. Transformation is eventually mentioned, later, but what was the transformation?

*Results*

251. I did not find Table 1 easy to understand, especially since its caption was just a heading, with no explanation . I gathered that it referred to total catches split up between all of the treatments, but I was confused that the total for BRR+VST was not the same as the total for colours, orientation and olfaction. It is hardly necessary to indicate that the percent of males was 100-X % when the percent of females is stated as X%.

254. The section to which the heading refers is not standardising the colours – it is comparing their efficacy.

256 (and all of the Results section). It would be much kinder on the reader if the results were tabulated, instead of presented as a mass of numbers in the text Because the Results are so stressful to read I have not read the section closely, so that there may be more things to correct than those mentioned below.

274. It is very confusing here, especially since it is indicated earlier that only one side of the board had a panel. What do the “two panels” mean – screens now put on two sides of the board, or two screens of differing colour put on one side, or ……..?

276. At many points the text could be more concise. Here is an example on shortening: “… the vehicle did not differ significantly.”

311. Comparison not standardisation.

324. indices not index.

*Discussion*

367. The results do CERTAINLY NOT contradict the indications of other reports that black induces relatively strong/rapid alighting responses. Indeed, the fact that the authors caught more flies on the black than the blue, when both colours were presented together suggests, as all other reports seem to do, that flies alight more readily on black as against blue. The authors seem to be muddled. Before musing further, they would do well to separate more clearly, in their own minds and the MS, the distinction between what attracts from a distance and what determines behaviour at close range. They should also consider that the truck itself is a huge competing surface for the attention of flies with the propensity to alight.

369. The notion that mobility affects the choice of colour on which to alight is certainly novel and intriguing, but it seems not to be supported by present results.

372 to 385. This is all somewhat airy-fairy since all mobile baits will move, so that flies cannot possibly alight on a mobile bait that does not move – such baits do not exist. Rather than getting into these imponderables it would be best to have much more data available, covering such things as the catches from baits of different size and colour, and different rates of movement.

393 to 406. This is all very unconvincing. It would become more credible if the authors explained how the habitat geometry translated into the apparent variation in catch distribution at baits. To try to help the authors I mused over the business myself, but found it difficult. This was especially so since, as the authors say, the flies mill around a screen extensively before alighting, so that whether they come from the wooded or open side of the road may have little effect on the particular side of the screen on which they alight.

410. I am not sure that the authors have interpreted Torr (1989) correctly. I would be amazed if Torr were not one of the reviewers so I will leave him to cover this business. In any event, there are several papers that indicate that close-range responses are affected substantially by odour. That said, I agree that odours tend to be more effective with stationary rather than mobile baits, mainly because the odours tend to be relatively ineffective for bringing flies to mobile baits from a distance.

415. Avoid split infinitive.

416. The VST as used by the authors was a stationary bait for much of the time. Maybe the odours from the VST attracted flies to the vicinity when the VST was stationary, and those flies promptly went to the VST when it started moving. If so, the author’s musings in the rest of the paragraph are undercut.

417. If I have understood the authors correctly, they are now saying that odours do affect close range behaviour, while previously seeming to say the opposite.

424. I think the authors might have overstated the business of habitat geometry in the present case, although it must be conceded that it could have some effect. Other factors could be involved, such as one species being studied in cool July and the other in warmer September. Perhaps some rephrasing is required if the authors wish to stick to the idea of habitat geometry being important. For example, as a quick stab, the drift might be: “Maybe the geometry of the habitats of the subspecies is sufficiently great to affect their responses to odour. Habit effects are considered to cause the differences between savannah and riverine species in the response to odour baits, but more needs to be done to assess what differences there might be between sub-species of G. morsitans. Meanwhile, we recommend baiting the VST ……… etc”

433. This is a quaint way of considering speed. Why not quote speeds in the normal way of km per hour. That would be most appropriate since the catch from mobile baits operated at speeds up to about 10-15km per hour depends mainly on the distance travelled rather the operating time *per se*. In contrast, catches at stationary baits depend more directly on the operating time.

442. Against this I suspect that many of the flies attracted to the VST might not alight, and that even the flies that do alight might not be retained by the sticky stuff. If the VST is to be recommended, it would have been good to quantify these matters. That would help in interpreting what the catches from the VST mean about the population being sampled.

443. Have the authors actually seen tsetse being blown off a bait by strong wind? I have not. Moreover, I suspect that if winds were that strong they would hinder flies arriving at the baits.

445. Have the authors got data to show that all flies that touch the sticky surface are permanently secured. In my experience, the sticky deposit is not that amazingly good.

446. The authors scoot over that fact that the VST requires a good road, whereas a BST can go along a rough path, and pick its way up a steep escarpment, among rocks and in marshy places where truck would bog down. Moreover, the BFR can catch flies in or between showers, but rain can upset the performance of sticky devices – and also the performances of electric traps, of course. These various benefits of the BFR are also shared with traps, with the added advantage that a trap works all day every day. I note that the authors acknowledge the accessibility problem later and say that it might be overcome by mounting sticky traps on motorbikes. I recall that this type of thing was tried in the Western Province of Zambia, albeit with electric traps not sticky traps on the bike, but that it did not find much favour by Luc Willemse and colleagues. I think the bikes were sent only where a truck could go.

452. Delete the full stop after hr.

452. The more rapidly a truck moves the more the dust and leaves it kicks up, and the more dust and debris there is swirling around the sticky deposit, to clog it and render it less effective.

453. Were most captured? Are actual data available to prove the point?

454. Smart modern phones will automatically record the speed and location at chosen intervals.

460. I will believe the story about flies not getting stuck-up in the present study. However, my general experience suggests that this might not hold when the stops are less frequent, the tracks are more bumpy, and the stick deposit is not very fresh. Do the authors have data for the proportion stuck-up under varying circumstances? I note the authors do, most sensibly, suggest that more research is needed on this matter if sticky traps are to be used extensively.

479. It is indeed appropriate to try to catch tsetse in places where it is required to improve our knowledge of the species present, and their abundance, condition and behaviour. Moreover, the authors have certainly indicated that a VST could be used to improve our knowledge. However, it remains far from clear whether a VST technique should be adopted as the standard, and equally unclear what sort of vehicle type/size/shape/colour/speed and operating plan should be adopted as the standard VST. In particular, the authors make little mention of the relative cost-effectiveness of the various sampling options available, and how that is affected by circumstances.

----------<O>----------
