# Supplementary material for: A novel vehicle-mounted sticky trap; an effective sampling tool for savannah tsetse flies Glossina morsitans morsitans Westwood and Glossina morsitans centralis Machado
Source: PLoS Negl Trop Dis. 2021 Jul 19;15(7):e0009620. doi: 10.1371/journal.pntd.0009620 (PMC8321396; doi:10.1371/journal.pntd.0009620)
Supplement: Attachment — Submitted filename: Responses to reveiwers_JM.docx [file pntd.0009620.s004.docx]

Reviewer#1 (Glyn Vale)

| Manuscript section | Line No. | Reviewer Comment | Response from authors | Action taken by authors |
| --- | --- | --- | --- | --- |
| Abstract | 26 | The BFR might have been the recommended mobile sampling system several decades ago, but very few people might recommend it for savannah tsetse today. | Other sampling tools are available to sample savannah tsetse. But in terms of cost-effective mobile sampling devices, BFR remains the most widely used technique | No action taken |
|  | 27 | If it is used, the BFR commonly allows for catches of tsetse from the ground and men. | When used as recommended by Robinson (1), the majority of flies tend to land on the screen rather than on the ground or on vegetation. Hence the ability to catch the on the screen is crucial | No action taken |
|  | 30 | Should the first mention of Glossina morsitans morsitans be written in full. Short for the next mentioned species is OK. | We agree with the reviewer | Changes are made in the text |
|  | 33 | The present MS deals with effectiveness not efficiency. Efficiency is best employed to refer to the catch as a proportion of what is available to be caught. | We agree with the reviewer | Changes are to reflect that the manuscript deals with effectiveness |
|  | 36 | If the term “catch index(ices)” is to be used, should the reference catch be made clearer. Thus, the reader might get the first impression that the reference is the BFR, but then it looks like maybe the reference is one of the screens. It gets especially confusing later when “index” gets mixed up with “times higher”. | We agree with the reviewer | Changes have been made in the text and the sentence now reads as follows: “Overall, the VST caught 2.42 (1.91 – 3.10; P<0.001) and 2.60 (1.50 – 3.21; P<0.001) times more G. m. centralis and G. m. morsitans respectively, than the BFR” (lines 41 – 43). |
|  | 36 | (Here and throughout). Would “transverse” be better than “perpendicular”. It could be considered that all screens were “perpendicular” in the sense of being upright. | We agree with the reviewer | Changes are made in the text |
|  | 38 | No significant effect. | We agree with the reviewer | Changes are made in the text |
|  | 41 | Comma respectively | We agree with the reviewer | Changes are made in the text |
|  | 43 | Delete “We conclude that” . | We agree with the reviewer | Changes are made in the text |
|  | 44 | is several times more effective for. | We agree with the reviewer | Changes are made in the text |
|  | 45 | Their = its. |  |  |
| Author Summary | 51 | The standardization aspect is not very clear. For example, you imply eventually that any of the colours tested will do, which tends to imply that standardization within the variables you tested is of little consequence. Perhaps the most important thing(s) to standardise are the size/shape/colour/speed of the vehicle – a matter to which you seem to have given no attention whatsoever. In particular, the vast majority of the surface of the mobile bait you used looks to have been the silverish truck. How does silver compete with blue or black Would the relative effectiveness of the screen colours change if the truck was, say, dull black or bright blue? | We agree with the reviewer. We have changed the title to reflect the scope of our study to “A Novel vehicle-mounted sticky trap; an effective sampling tool for savannah tsetse flies *Glossina morsitans morsitans* Westwood and *Glossina morsitans centralis* Machado” We have removed the word standardization from the text throughout the manuscript and added in the discussion a paragraph that discusses the limitations of the study in terms of standardization of size/shape/colour and speed of vehicle. | Changes in text made lines 1 – 3 |
|  | 52 |  |  |  |
|  | 53 |  |  |  |
|  | 54 |  |  |  |
|  | 55 |  |  |  |
| Introduction | 72 | Strictly speaking the genus Trypanosoma is not the causative agent. Instead, it is a few of the species within that large genus. | We agree with the reviewer. | The manuscript has been revised removing “genus Trypanosoma” |
|  | 75 | 30, 000 per year in 19?? 000 to >1000 in 20??. | We agree with the reviewer | Years are added in the text |
|  | 76 | AAT is a significant cause | We agree with the reviewer | The change is made in the text |
|  | 77 | Estimated to cause three | We agree with the reviewer | The change is made in the text |
|  | 84 | G. m. morsitans. | We agree with the reviewer | The change is made in the text |
|  | 86 | You seem not to have indicated that nagana = AAT. | This is incorrect. AAT is indicated to be nagana in line 73. | No action taken |
|  | 92 | Cost-effective tools for sampling tsetse are …… Bear in mind that vehicles and fuel are pricey. | We agree with the reviewer. We have removed evaluation of all aspects of cost effectiveness of the VST as it was beyond the scope of the study | The change has been made in the text lines 117 – 122. |
|  | 96 | “stimuli” not “responses”. | We agree with the reviewer | Responses changed to stimuli |
|  | 97 | Delete “primarily”? | We agree with the reviewer | Primarily deleted |
|  | 99 | the most attractive from a distance, whereas…. Delete comma at end of line. | We agree with the reviewer | The change is made in the text |
|  | 100 | The attractiveness of shapes increases in the order ……. | We agree with the reviewer | The change is made in the text |
|  | 101 |  |  |  |
|  | 102 | Is “consequently” right here. If certain settings for various features are shown to result in greatest catches, the fact that traps with a range of settings are used is more of a surprise than a consequence. | We agree with the reviewer | The change is made in the text |
|  | 107 | Delete “Volatile”. All olfactory cues are volatile. Remove the split infinitive. Quantify the range of what is meant by “dramatic”. | We agree with the reviewer | The change is made in the text |
|  | 109 | . Delete “other”. | We agree with the reviewer | The change is made in the text |
|  | 111 | their relatively low cost. If you are worried about cost, why not say that cow urine can replace the expensive phenols? | Acquiring sufficient cow urine depends on the good will of farmers. This may not always be forth coming and hence the need for synthetic attractants. Furthermore, storage of large quantities of cow urine is impractical. | The word relative is included in the sentence. |
|  | 116 | Traps give a good idea of daily activity patterns because flies must be very active to visit the traps. Against this, mobile devices go the flies, which then need to do little more than flop off their resting site to arrive at passing baits. A single trap might catch less than a vehicle mounted trap per unit time, but many traps can be bought for the cost/hire of a truck, and traps can be left in place for several days or weeks before needing human attention. Dr William Shereni, in Zimbabwe, arranges his traps for inspection at intervals of up to 3- months. How many vehicle-hours or miles would be needed to catch as many flies as, say, a set of a dozen odour-baited traps operated for a month or so? | We agree that traps give a good idea of daily activity patterns because flies must be very active to encounter the traps. However,  Kuzoes and Schofield (2), Leak et al (3), Krafsur (2009) and Bouyer et al (4), all indicate that deploying traps over large areas is expensive. The advantage Dr. Shereni has in Zimbabwe is that survey teams are permanently stationed in close proximity to the major tsetse belt in the north of the country. In such a situation the cost of team deployment is not significant. However, in a situation like that which prevails in Zambia, no permanent teams are stationed near any of the eight major belts. Tsetse surveys depend on mobilising teams of 30 personnel and having them camp in an area for 15 – 20 days. The cost of deployment in these circumstances is very high. Further, in a survey to determine presence and absence, a trap needs to be stationed at a site for a minimum of three days. This not only increases the cost but limits geographical coverage. Therefore, a tool that can rapidly detect tsetse flies over a transect of minimum 80 km/day is more desirable in such surveys than stationary traps | No action is taken |
|  | 118 | This is a meaningless statistic as stated here. Does it refer to $283 per 1 trap per 1sqkm per day – or what? For sampling, one worries somewhat more about the cost per sampled fly of the sex/species/age/physiological state that is pertinent | We agree with the reviewer. However, as stated by Leak et al (3) and Bouyer et al (4), deploying traps over large areas is expensive. | The statistic has been removed. |
|  | 119 | The use of traps can hardly be “prohibitive” since it has long been the standard system operated by a severely cash-strapped Zimbabwe. | See comments for 116 above. | Leak et al (3) and Bouyer et al. (4) are added as a reference to validate the point. |
|  | 119 | . Has the sampling of tsetse really been geographically restricted? I suspect that tsetse have been sampled over areas greater than those sampled for most, even any, mosquito species/subspecies. I suspect also that the cost of genetic analysis of a tsetse might be effectively several times more pricey than actually getting the fly. Are geneticists or other students of fly biology worried about getting flies that are “stuck up”? | Krafsur (5), Bouyer et al. (4) and Krafsur and Maudlin (6) have all highlighted limited geographic sampling as a major limitation to tsetse population studies. While it is true that tsetse flies have been widely sampled, when you compare this to their geographical range, this sampling has been inadequate to accurate describe their population genetics. | No action taken |
|  | 120 |  |  |  |
|  | 122 | Use “translocation”. “Movement” can mean no more than a little flapping/wobbling of a screen, which is known not to be particularly attractive. Remove the split infinitive. | We agree with the reviewer | The change is made in the text |
|  | 123 | In general, a mobile bait is more effective than a stationary bait for catching G. m. However, it depends on what sort of mobile bait you are using, what sort of stationary one, and the condition of the fly you are after. | We agree with the reviewer | No action taken |
|  | 124 |  |  |  |
|  | 125 | vehicle-mounted electric taps ….. | We agree with the reviewer | The change is made in the text |
|  | 126 | The VET cannot be desperately impractical if it has been used widely and long for sampling in impoverished Zimbabwe. | We agree with the reviewer | We agree with the reviewer |
|  | 131 | That is indeed true, provided the mobile device you develop is indeed more COST-EFFECTIVE than any other mobile or stationary device, and provided you demonstrate its superiority in that regard. | We agree with the reviewer | No action taken |
|  | 135 | The BFR might be standard in Zambia, but it is widely believed to be somewhat backward for savannah tsetse | The BFR currently operated in Zambia is that developed by Robinson (1). |  |
| Materials and methods | 144 | Delete “located”. | We agree with the reviewer | The change is made in the text |
|  | 145 | Does the reader really care much about the climatic details. Indeed, will the reader pounce with much eagerness on Fig1 and the details of the Zambian distribution? Are these things just “packing”? | We agree that climate details may not be very important for the text. However, the details shown in Fig 1 are there to impress on the reader that vast area were the VST can be used. | Climate details removed |
|  | 156 | Conducting the study over a mere nine days is a bit thin. Presumably, July was sunny and cold. The other work was conducted for nine days in September. It would have been sunny but warmer then. Is there any reason to suspect seasonal effects -- perhaps when clouds alter the illumination of the screens? Was there a difference in catch between the predominantly sunny and predominantly shady sides of the screen? | The fact that the study at each locality could be conducted in 9 days speaks to the effectiveness of the tool. As shown in the results section, over the 18-day period, over 11000 tsetse were collected. A quick literature search will indicate that such an output is generally reported for studies longer than 2 years using stationary traps. Further, a look at the results shows remarkable consistence in all but one experiment despite studying two different sub-species in two different seasons. Thus, it is unlikely that seasonal effects would alter the results significantly. | No action taken |
|  | 170 | You will get in the bad books of Vestergaard Frandsen if you do not spell the name correctly. Did you rally deal with the Danish, as against the Swiss, arm of the company? | We agree with the reviewer | The change is made in the text |
|  | 171 | As stated here, the screen was covered with cloth on one side, but had no cloth on the other. Is that really right? | Yes, this is correct. However, to construct a two-sided sticky trap, two panels were fixed on either side of the steel frame. This has been clarified in the text. | The clarification has been made in the text |
|  | 172 | “strip” not “stripe”? I did not see mention of how long the sticky film was used before replacement. How often must it be replaced under various operating conditions? | Sticky film was replaced at the start of each experiment, thus every 2 – 3 days. This has been highlighted in the text. The study did not investigate how often the film needs to be replaced. However, the product is design to operate in all weather conditions. | The change is made in the text |
|  | 182 | I am nervous of this reflectance business since there is the plain fact shown, by field experiments, that tsetse are reluctant to alight on shiny surfaces -- and sticky surfaces are shiny. How do the authors talk their way around this? The reactions of a tsetse are more directly pertinent to the present case than the outputs of a spectrometer or any other machine. | Experiments have indeed shown that tsetse are reluctant to land on shiny surfaces. The component of light responsible for this observed phenomenon is light in the ultraviolet spectrum (7–9). These researchers also showed that painting surfaces with 2,4-dihydroxybenzophenone, selectively eliminates ultraviolet wavelength making colour perception at 450 nm sharper. The adhesive films used in this study are coated with a UV absorber hence this component of light is not reflected, as verified by spectrophotometric measurements as highlighted in the manuscript. Therefore, what may be perceived as a shiny surface buy the human eye, is not shiny at all to the tsetse fly. | No action taken |
|  | 198 | The sentence here is too long and complex, and is not helped by the string of confusing adjectives/nouns in “best panel trap colour design”. | We agree with the reviewer | The change is made in the text |
|  | 203 | (here and elsewhere). k not K, in km. | We agree with the reviewer | The change is made in the text |
|  | 206 to 236 | Many of the descriptions given here tend to be very confusing. For example, is it the truck or the BFR that went along the transects? It seems from Fig 2 that the transects were much longer than 300m. Much time was spent waiting, and the VST catching was performed only over 300m. Hence, how much of the catch was represented by flies that visited the stationary truck and got caught when the vehicle moved off? Is that really the way that a VST would be operated in normal surveys? The placement of odour attractants described might have been used by Willemse but I am not sure that it should be regarded as “standard”. | We agree with the reviewer | This entire section has been re-written to provide more clarity with inclusion of the reviewer’s recommendations. As highlighted, the overall design was the same, in each experiment, differing only in the treatments used. |
|  | 214 | “Vehicle fly round” is a term new to this MS. Does it mean a VST. | The term is supposed to describe the operation of the VST. However, to improve clarity the term is removed from the text. | The term has been removed from the text |
|  | 222 | “Speciate” means to evolve into a distinct species. | We agree with the reviewer. Identification is used. | The change is made in the text |
|  | 231 & 236 | Why use “tsetse” in some places and “tsetse fly” in others. The former is preferable, being more concise. | We agree with the reviewer | The change is made in the text |
|  | 238 | The stats section is skimpy. I would prefer more details of what was actually done by the packages. For example, were the catches transformed for analysis. Transformation is eventually mentioned, later, but what was the transformation? | We agree with the reviewer | A more detailed description of the statistics used is given. |
| Results | 251 | I did not find Table 1 easy to understand, especially since its caption was just a heading, with no explanation . I gathered that it referred to total catches split up between all of the treatments, but I was confused that the total for BRR+VST was not the same as the total for colours, orientation and olfaction. It is hardly necessary to indicate that the percent of males was 100-X % when the percent of females is stated as X%. | We agree with the reviewer | The table has been revised as recommended and the caption improved. |
|  | 254 | The section to which the heading refers is not standardising the colours – it is comparing their efficacy. | We agree with the reviewer | The change is made in the text |
|  | 256 | (and all of the Results section). It would be much kinder on the reader if the results were tabulated, instead of presented as a mass of numbers in the text Because the Results are so stressful to read I have not read the section closely, so that there may be more things to correct than those mentioned below. | We agree with the reviewer | The entire results section has been re-written as recommended by the reviewer. The major experimental results are now tabulated in two tables, one for each subspecies. |
|  | 274 | It is very confusing here, especially since it is indicated earlier that only one side of the board had a panel. What do the “two panels” mean – screens now put on two sides of the board, or two screens of differing colour put on one side, or ……..? | This point has been clarified in the methods section. Two panels mean, one panel placed on either side of the steel frame. The choice of colour is determined by the colour comparison experiment. Thus, in a two-sided sticky trap panel, two panels of the same colour are placed on either side of the steel frame. | Clarity has been made in the methods section. |
|  | 276 | At many points the text could be more concise. Here is an example on shortening: “… the vehicle did not differ significantly.” | We agree with the reviewer | The change is made in the text, as above. |
|  | 311 | Comparison not standardisation. | We agree with the reviewer | The change is made in the text |
|  | 324 | indices not index. | We agree with the reviewer | The change is made in the text |
| Discussion | 367 | The results do CERTAINLY NOT contradict the indications of other reports that black induces relatively strong/rapid alighting responses. Indeed, the fact that the authors caught more flies on the black than the blue, when both colours were presented together suggests, as all other reports seem to do, that flies alight more readily on black as against blue. The authors seem to be muddled. Before musing further, they would do well to separate more clearly, in their own minds and the MS, the distinction between what attracts from a distance and what determines behaviour at close range. They should also consider that the truck itself is a huge competing surface for the attention of flies with the propensity to alight. | We agree with the reviewer. The arguments presented here need further clarification and refinement. The results indicated that the colours used had equal attraction and landing differences were not observed when the colours were used singularly (8). However, when blue and black were present together, landing was predominantly on the black (10). Thus, the results do not contradict literature as correctly highlighted by the reviewer. This was initially misrepresented. Further, tsetse visual orientation to a host occurs in close proximity. All the above points have been clarified and corrected in the manuscript. Despite the truck being a huge competing factor for tsetse with the propensity to alight, it was a constant in all the experiments undertaken and therefore does not alter the observations. | The argument has been clarified taking into account the recommendations of the reviewer. |
|  | 369 | The notion that mobility affects the choice of colour on which to alight is certainly novel and intriguing, but it seems not to be supported by present results. | We agree with the reviewer | A recommendation stating the need to generate more data to validate this point is included. |
|  | 372 to 385 | This is all somewhat airy-fairy since all mobile baits will move, so that flies cannot possibly alight on a mobile bait that does not move – such baits do not exist. Rather than getting into these imponderables it would be best to have much more data available, covering such things as the catches from baits of different size and colour, and different rates of movement. | As stated above in 367, the arguments have been reframed in line with the recommendation of the reviewed. We further agree that the size effect and rate of movement of panels needs to be investigated. | Changes are made in the text accordingly, calling more attention to research that needs to be undertaken in the future. |
|  | 393 to 406 | This is all very unconvincing. It would become more credible if the authors explained how the habitat geometry translated into the apparent variation in catch distribution at baits. To try to help the authors I mused over the business myself, but found it difficult. This was especially so since, as the authors say, the flies mill around a screen extensively before alighting, so that whether they come from the wooded or open side of the road may have little effect on the particular side of the screen on which they alight | Large dambo areas lacking sufficient tree cover are known to be inhospitable to savannah tsetse (11). Therefore, we do not expect that the dambo area would be able to supply an equivalent amount of individual tsetse as the wooded area. We do however agree the that flies mill around a screen before landing. Having this in mind, we argue that the propensity to circulate towards the inhospitable side of the screen compared to the favourable side, should be low. Tsetse flies will tend to stay and fly in favourable environments and given the choice of attacking a bait from the hospital or inhospitable side, it is more like that the former approach will be favoured. | An explanation on how habitat geometry is affecting availability of flies at a bait is given. |
|  | 410 | I am not sure that the authors have interpreted Torr (1989) correctly. I would be amazed if Torr were not one of the reviewers so I will leave him to cover this business. In any event, there are several papers that indicate that close-range responses are affected substantially by odour. That said, I agree that odours tend to be more effective with stationary rather than mobile baits, mainly because the odours tend to be relatively ineffective for bringing flies to mobile baits from a distance. | Torr (12) found that only carbon dioxide affected landing response, and many papers have validated this. The synthetic odours used in this study have generally been shown to have no effect on landing. However, we agree that the reference given here may mislead some readers. Therefore, it is removed. | Reference is removed. |
|  | 415 | Avoid split infinitive | We agree with the reviewer | The change is made in the text |
|  | 416 | The VST as used by the authors was a stationary bait for much of the time. Maybe the odours from the VST attracted flies to the vicinity when the VST was stationary, and those flies promptly went to the VST when it started moving. If so, the author’s musings in the rest of the paragraph are undercut. | This point has been clarified in the methods section of the manuscript. Immediately after completing a traverse, the odours were sealed in an air tight container. Thus, when milking of the trap, its 300 m movement to the next transects and during the waiting period, no odours were dispensed. | No action taken |
|  | 417 | If I have understood the authors correctly, they are now saying that odours do affect close range behaviour, while previously seeming to say the opposite. | What we are highlighting here is that odours affect *G. m. morsitans* and *G. m. centralis*, differently. In the former, close range odour have no effect, while in the later our results suggest an effect. Note that there is very little literature on odour mediated host location in *G. m. centralis*. | No action taken |
|  | 424 | I think the authors might have overstated the business of habitat geometry in the present case, although it must be conceded that it could have some effect. Other factors could be involved, such as one species being studied in cool July and the other in warmer September. Perhaps some rephrasing is required if the authors wish to stick to the idea of habitat geometry being important. For example, as a quick stab, the drift might be: “Maybe the geometry of the habitats of the subspecies is sufficiently great to affect their responses to odour. Habit effects are considered to cause the differences between savannah and riverine species in the response to odour baits, but more needs to be done to assess what differences there might be between sub-species of G. morsitans. Meanwhile, we recommend baiting the VST ……… etc” | We agree with the reviewer. | Changes are made in the text as suggested. |
|  | 433 | This is a quaint way of considering speed. Why not quote speeds in the normal way of km per hour. That would be most appropriate since the catch from mobile baits operated at speeds up to about 10-15km per hour depends mainly on the distance travelled rather the operating time per se. In contrast, catches at stationary baits depend more directly on the operating time. | We agree with the reviewer | The change is made in the text |
|  | 422 | Have the authors actually seen tsetse being blown off a bait by strong wind? I have not. Moreover, I suspect that if winds were that strong they would hinder flies arriving at the baits. | We agree with the reviewer | The sentence is deleted. |
|  | 445 | Have the authors got data to show that all flies that touch the sticky surface are permanently secured. In my experience, the sticky deposit is not that amazingly good. | The Rentokil adhesive film used is this study suggest that it is reasonable to assume that all flies that touch the sticky surface are permanently secured. In fact, the authors have observed flies on the sticky screens 3 days after capture, despite moving at normal speed from the camp to an office a 100 km away. | No action taken |
|  | 446 | The authors scoot over that fact that the VST requires a good road, whereas a BST can go along a rough path, and pick its way up a steep escarpment, among rocks and in marshy places where truck would bog down. Moreover, the BFR can catch flies in or between showers, but rain can upset the performance of sticky devices – and also the performances of electric traps, of course. These various benefits of the BFR are also shared with traps, with the added advantage that a trap works all day every day. I note that the authors acknowledge the accessibility problem later and say that it might be overcome by mounting sticky traps on motorbikes. I recall that this type of thing was tried in the Western Province of Zambia, albeit with electric traps not sticky traps on the bike, but that it did not find much favour by Luc Willemse and colleagues. I think the bikes were sent only where a truck could go. | These limitations are discussed further down in the manuscript | No action taken |
|  | 452 | Delete the full stop after hr. | We agree with the reviewer | The change is made in the text |
|  | 452 | The more rapidly a truck moves the more the dust and leaves it kicks up, and the more dust and debris there is swirling around the sticky deposit, to clog it and render it less effective. | This is reasonable. In our experience however, at 20km/hr, the sticky film can be operated successfully. | No action taken |
|  | 453 | Were most captured? Are actual data available to prove the point? | Most are captured. | The change is made in the text |
|  | 454 | Smart modern phones will automatically record the speed and location at chosen intervals | We agree with the reviewer. | No action is taken |
|  | 460 | I will believe the story about flies not getting stuck-up in the present study. However, my general experience suggests that this might not hold when the stops are less frequent, the tracks are more bumpy, and the stick deposit is not very fresh. Do the authors have data for the proportion stuck-up under varying circumstances? I note the authors do, most sensibly, suggest that more research is needed on this matter if sticky traps are to be used extensively. | Data collected thus far seems to suggest that a reasonable proportion of tsetse flies land on the screen using their feet. However, as highlighted by the reviewer and clearly pointed out in the Manuscript, more work needs to be done to evaluate this point. | No action taken |
|  | 479 | It is indeed appropriate to try to catch tsetse in places where it is required to improve our knowledge of the species present, and their abundance, condition and behaviour. Moreover, the authors have certainly indicated that a VST could be used to improve our knowledge. However, it remains far from clear whether a VST technique should be adopted as the standard, and equally unclear what sort of vehicle type/size/shape/colour/speed and operating plan should be adopted as the standard VST. In particular, the authors make little mention of the relative cost-effectiveness of the various sampling options available, and how that is affected by circumstances. | We have clarified the scope of our study as an evaluation of VST as an effective mobile sampling tool for tsetse flies. We have also recommended for further studies that will focus on standardization and cost-effective determination | The change is made in the text |

Reviewer # 2

| S/N | Key Review Criteria | Reviewer Comment | Response from authors | Action taken by authors |
| --- | --- | --- | --- | --- |
| 1 | Are the objectives of the study clearly articulated with a clear testable hypothesis stated? | Yes, the authors state that they aim to evaluate a vehicle-based sampling method against the 'traditional' fly round. The Introduction misrepresents some of the disadvantages of traps. The cost estimate is not for sampling but for control operations. Many traps can be deployed by a team and the trap can operate without humans being present whereas a fly round requires humans and possibly cattle to be present. A survey using a vehicle has all the costs of operating a vehicle. Nonetheless, there is merit in exploring better mobile sampling methods for *G. morsitans* subspecies. | Leak et al (3) highlights the high cost of conducting surveys using traps. We however agree that the cost of using traps for control rather than sampling survey is what is quoted in the text. Where permanent teams are deployed close to survey areas, traps may have significant advantages. However, if survey teams require frequent deployment at high cost, the ability to rapidly obtained a useful sample over a large area can only be done using mobile devices | The cost estimate for the traps has been removed from the manuscript. |
| 2 | Is the study design appropriate to address the stated objectives? | I found the description of the experimental design very difficult to follow. It seems that the authors had a number of different transects which they operated simultaneously but it is not clear how they randomised the allocation of sampling methods between transects. In general, the language is not very clear throughout the manuscript. I would expect to see some form or Latin square design of transects x days x sampling methods. Are 'blocks' equivalent to transects? I think a clearer description of the experimental design would help with a graphic of the design in the SI perhaps | We agree with the observation that the section on experimental design was difficult to follow. It has been rewritten to improve clarity.  Use of Latin Square Design was deemed inappropriate as the assumption of lack of interaction of factors could not be reasonably disqualified. The completely randomised block design was however appropriate as site (transect) and time of sampling variation was adequately blocked. | The entire section on experimental design has been rewritten, to improve clarity. |
| 3 | Is the population clearly described and appropriate for the hypothesis being tested? | The study sites are well described and the catches are adequate for comparing the different sampling methods. | We agree with the reviewer. | No action taken |
| 4 | Is the sample size sufficient to ensure adequate power to address the hypothesis being tested? | Yes, the catches of tsetse are sufficient to test the hypotheses. However, each of the experiments was conducted over only 2-3 days. For instance, the comparison of the fly round and VST was conducted over two days only. It is unclear to me whether treatments have been truly randomised and that 'blocks' are independent. | The fact that adequate samples were collected in the stated time speaks to the superior effectiveness of the VST compared to the traditional methods that require more operational time.  Different blocks constituted 1 km transects, set 300 m apart (13), with groups of adjacent days as experimental units (14). Treatments were randomly allocated to days within blocks (S1) such that each transect was traversed at the same time, in consecutive days. Thus, site and time of sampling variation was blocked.  Inspection of block means clearly shows that blocks were independent. | The entire section on experimental design has been rewritten, to improve clarity. |
| 5 | Were correct statistical analysis used to support conclusions? | The analyses are poorly described. Did you use a glm or glmm? How did you account for days, transects and treatments? The data for all the experiments are contained in Table 1 but the results of statistical analyses are not presented here. The analyses are presented in verbose and repetitive text. I would much prefer to see these presented in a table with mean catches, CIs and contrasts. The authors refer to a catch index but this is not explained in the methods. | We agree with the observations of the reviewer. | The entire section on statistical analysis is rewritten. The structure formula for the model is presented detailing how the block and day effects are separated from treatment means. |
| 6 | Are there concerns about ethical or regulatory requirements being met? | The study meets all the expected ethical and regulatory requirements. | We agree with the reviewer | No action taken |
| 7 | Does the analysis presented match the analysis plan? | Probably, but the presentation is very difficult to follow. I would prefer to see the mean catches, CIs, catch indices and P values presented in a Table. | We note the reviewer concerns | The entire results section has been rewritten and numerical results presented in two tables, one for each subspecies. |
| 8 | Are the results clearly and completely presented? | No. See above. The results need to be presented in a Table or figure rather than pages of text. | We note the reviewer concerns | The entire results section has been rewritten and numerical results presented in two tables, one for each subspecies. |
| 9 | Are the figures (Tables, Images) of sufficient quality for clarity? | No. There is a single Table of the results which presents total catches. I would like to see the mean catches, CIs, catch indices and P values presented in a Table and the text comments on the main findings. | We note the reviewer concerns | The entire results section has been rewritten and numerical results presented in two tables, one for each subspecies. |
| 10 | Are the conclusions supported by the data presented? | The data suggest that tsetse can be caught on vehicle-mounted panels, and that the vehicle mounted devices catch more tsetse than a traditional fly round. The results (6 pages) and discussion are verbose (6 pages) for findings that could be clearly and succinctly presented in a single table. | We note the reviewer concerns | The entire results section has been rewritten and numerical results presented in two tables, one for each subspecies. |
| 11 | Are the limitations of analysis clearly described? | There is discussion of the limitations of the method but the authors ignore the costs of using a vehicle to monitor tsetse compared to use of traps. | Evaluation of cost-effectiveness of the VST relative to other sampling tools was beyond the scope of the study. This has been made clear in the text and we recommend further studies that will investigate this. | Clarity on the scope of the study has been made. |
| 12 | Do the authors discuss how these data can be helpful to advance our understanding of the topic under study? | The authors suggest that vehicle-counted traps could be used to monitor G. morsitans in tsetse control operations. | We agree with the reviewer | No action taken |
| 13 | Is public health relevance addressed? | Yes, the authors relate their aims and findings to the control of African trypanosomiasis | We agree with the reviewer | No action taken |
| 14 | Summary and General Comments | The manuscript is verbose and rambling and the language unclear. I strongly suggest that the authors reduce the overall length, improve the presentation of results and the text highlights the main findings | We have improved the manuscript | Changes have been made in the manuscript |

References

1.	Robinson TP. Tsetse Surveys: Field Manual. Insect Pest Management Initiative: Zambia; 1995.

2.	Kuzoes FAS, Schofield CJ. Strategic Review of Traps and Targets for Tsetse and African Trypanosomiasis Control. Geneva; 2005.

3.	Leak SG., Ejigu D, Vreysen MJB. Collection of Entomolological Baseline Data for Tsetse Area-Wide Integrated Pest Management Programmes. Rome: FAO/IAEA; 2008.

4.	Bouyer J, Dicko AH, Cecchi G, Ravel S, Guerrini L, Solano P. Mapping landscape friction to locate isolated tsetse populations that are candidates for elimination. PNAS. 2015;112(47):14575–80.

5.	Krafsur ES. Tsetse flies: Genetics, evolution, and role as vectors. Infect Genet Evol. 2009;9(1):124–41.

6.	Krafsur ES, Maudlin I. Infection , Genetics and Evolution Tsetse fl y evolution , genetics and the trypanosomiases - A review. Infect Genet Evol. 2018;64:185–206.

7.	Green CH, Cosens D. Spectral responses of the tsetse fly, *Glossina morsitans morsitans*. J Insect Physiol. 1983;29(10):795–800.

8.	Green CH, Flint S. An analysis of colour effects in the performance of the F2 trap against *Glossina pallidipes* Austen and *G . morsitans morsitans* Westwood ( Diptera : Glossinidae ). Bull Entomol Res. 1986;76:409–18.

9.	Green CH. The effect of colour on trap- and screen-orientated responses in *Glossina palpatis palpalis* ( Robineau-Desvoidy ) ( Diptera : Glossinidae ). Bull Entomol Res. 1988;78:591–604.

10.	Green CH. Bait methods for tsetse control. Adv Parasitol. 1994;34:229–91.

11.	Vreysen MJB, Seck MT, Sall B, Bouyer J. Tsetse flies: Their biology and control using area-wide integrated pest management approaches. J Invertebr Pathol [Internet]. 2013;112(SUPPL.1):S15–25. Available from: http://dx.doi.org/10.1016/j.jip.2012.07.026

12.	Torr SJ. The host‐orientated behaviour of tsetse flies (Glossina): the interaction of visual and olfactory stimuli. Physiol Entomol. 1989;14(3):325–40.

13.	Vale GA, Phelps RJ. Sampling problems with tsetse flies ( Diptera: Glossinidae). J Appl Ecol. 1978;15(3):715–26.

14.	Torr SJ. Dose responses of tsetse flies (Glossina) to carbon dioxide, acetone and octenol in the field. Physiol Entomol. 1990;(15):93–103.
