# Supplementary material for: A novel vehicle-mounted sticky trap; an effective sampling tool for savannah tsetse flies Glossina morsitans morsitans Westwood and Glossina morsitans centralis Machado
Source: PLoS Negl Trop Dis. 2021 Jul 19;15(7):e0009620. doi: 10.1371/journal.pntd.0009620 (PMC8321396; doi:10.1371/journal.pntd.0009620)
Supplement: Attachment — Submitted filename: Responses to reveiwers_JM_2.docx [file pntd.0009620.s005.docx]

Reviewer#1

|  | Line No. | Reviewer Comment | Response from authors | Action taken by authors |
| --- | --- | --- | --- | --- |
| Divergent Opinions | 145 | The clearest, simplest and most concise means of impressing the reader with the extent of the infestations is to indicate in the text the sqkm covered – as indeed the authors have already done. Heaven help us and our readers if every time an important insect is discussed it is necessary to produce also a map of where the creature occurs. Likewise, in most papers we hardly need a map of where behavioural studies were performed – a line of text and a map reference would do. Moreover, the maps of the meandering paths used in each study area do not tell the reader much. It would have been more interesting to have shown the vegetation along the transects, especially since the authors indicate that the vegetation is not homogeneous in some places. Hence, while agreeing to the continued inclusion of the maps, I maintain my opinion that their main benefit is that of adding a bit more colour and packing to an otherwise skimpy MS. | The reviewer makes a compelling argument. While we were of the view that the Map inclusion could provide context to the extent of the problem, we concede that the concise manner in which to indicate the extent of infestation is as advised by the reviewer. | Both maps are removed from the Manuscript. |
|  | 393 to 406. | The authors’ proposition seems to be that tsetse will not fly in the direction of unfavourable habitat despite having a bait to explore. That proposition is difficult to square with the fact that tsetse are shown to visit a bait that is travelling in an inhospitable area along the edge of good woodland habitat. It is a pity that the authors made no serious attempt to substantiate this proposition experimentally. The catches from a screen coloured on both sides, with sticky deposit on both the woodland and dambo sides, or on each side separately, might have been pursued more fully to add greater substance and interest to the MS. | We agree with the reviewer. Our results do not provide evidence to support our claim that habitat influenced the availability of flies on the panels. | This argument has been removed from the manuscript. The difference in response is maintained and a recommendation to further investigate the cause is inserted. |
|  | 445 | (Perfect sticky deposit?) – The authors seem to claim that because the film they used was a sticky product of Rentokil it is reasonable to assume that all flies that touch the screen get caught. This deserves some supporting evidence, but none is offered. Instead, all the reader gets is the anecdotal and unsurprising indication that if a fly gets well stuck it remains stuck. The point at issue is how well the fly gets stuck at the first touch. | The reviewer rightfully points out that no experimental evidence is given by the claim that Rentokil sticky film capture all flies that touch the screen. We agree with the reviewer that this requires further investigation to validate the claim | The statement is deleted. We however highlight that the catch size of the VST is consistent within the effective range of the sticky film. |
|  | 453 | (Further praise of sticky deposits) – The authors have not answered the question I posed. They state, simply and baldly, that “Most were captured”. Maybe they have no answer to offer. | We agree with the reviewer as no experimental evidence is available to support this claim. | The statement is deleted from the text. |
| Specific comments on the revised MS | Line No. | Reviewer Comment | Response from authors | Action taken by authors |
| Introduction | 32 | Delete “fly” | We agree with the reviewer | Fly deleted |
|  | 34 | Zambia comma | We agree with the reviewer | Comma included |
|  | 39 | differences; “or” not “and”? | We agree with the reviewer | Sentence revised and now reads ‘Attractants did not significantly increase VST catch index for *G. m. morsitans*’ |
|  | 45 | respectively comma | We agree with the reviewer | Sentence revised accordingly |
|  | 50 | insert “m.” in G. m. morsitans, and italicize the name. Is the reader to believe that the VST is used to sample the BFR? | We agree with the reviewer | Sentence revised to give more clarity |
|  | 54 | G. m. morsitans | We agree with the reviewer | Change made |
|  | 56 | “its” not “theirs”. | We agree with the reviewer | Change made |
|  | 91 | G. m. morsitans. | We agree with the reviewer | Change made |
|  | 95 | Delete “Map showing the “. We can see that it is a map. | We agree with the reviewer | Entire caption has been deleted as the Map is removed from the text |
|  | 106 | Delete second comma | We agree with the reviewer | Change made |
|  | 118 | “lower” implies “relative”. If you must use “relative”, what about: “relatively low cost”? | We agree with the reviewer | Change made |
|  | 120 | tsetse’s. delete “fly”. | We agree with the reviewer | Change made |
|  | 123 | – It is strange to say that a device which samples activity can misrepresent activity patterns. It would be good to give a direct reference to the point, rather than the reviews offered. | We agree with the reviewer | Reference is added |
|  | 123 | Split infinitive. | We agree with the reviewer | Change is made |
|  | 125 | often makes their use prohibitive. You cannot imply that the use is always prohibitive when, in reality, the traps are often used for large-scale surveys | We agree with the reviewer | The sentence has been revised and now reads’ may limit their use in large scale surveys’. |
| Materials and methods – It appears that the reviewer used the previous version to review part of this section. However, certain concerns he raises are still valid and are addressed. | 140 | morsitans comma, particularly AT low population (No S) densities | We agree with the reviewer | Change made |
|  | 141 | Delete “set out to”. | We agree with the reviewer | Deletion is made |
|  | 142 | delete first “and”. | We agree with the reviewer | Change made |
|  | 143-144 | to identify the optimal colour and orientation of the trap and to assess | We agree with the reviewer | Change made |
|  | 145 | Why keep giving the name in full, together with its acronym, when you have already explained the acronym? | We agree with the reviewer | Change made |
|  | 146 | The same type of booboo as above is made again in respect of the BFR | We agree with the reviewer | Change made |
|  | 159 | Glossina m. morsitans | We agree with the reviewer | Change made |
|  | 164-165 and 174-175 | in each case a reference to the diet would not be amiss. | We agree with the reviewer | References are made |
|  | 166 and 176 | km | The change was made in the fist revision in the entire manuscript. | The manuscript was re-checked to ensure this correction was affected in all sections. |
|  | 178 | Two fabrics were used in trap construction. These were | We agree with the reviewer | Change made |
|  | 180 | They were cut | We agree with the reviewer | Change made |
|  | 190-196 | I doubt the story offered here. The spectral reflectance of the cloth might not be affected much by the film, but the point at issue is the reflectance of the film. Maybe, as the authors say, the film absorbs UV light, and maybe the cloth absorbs all or most of the non-UV light that gets through the film, but that says little about the non-UV light that is reflected by the film. Fig. 3 shows clearly that white light visible to humans is reflected by the film, and such light contains wavelengths known to be perceptible to tsetse. The upshot is that the light coming from the black and/or blue panels coated with film is not the same as the light from panels that are not so coated. | From the results section, the reflectance of the film appears not to affect the performance of the panels. Byamungu et al. (1), Oloo et al. (2) and Mbewe et al (3) have successfully used these films to enumerate catches on stationary traps. | No action taken |
|  | 197 | Butanone and 1-octen-3-ol were used as attractants, according to …. | We agree with the reviewer | Change made |
|  | 208 | “completely randomized block design experiments” is about as ugly as it gets. | We agree with the reviewer | The word completely is deleted |
|  | 209 | In the same vein, “best panel trap colour design” is vague and stressful. For example, does it mean the colour of the best panel trap, or the best colour of the panel trap. The whole sentence needs reorganizing and I hope I can leave that to persons who will know better than I what the sentence is trying to say. | We agree with the reviewer | Sentence has been revised |
|  | 212 | outlined | We agree with the reviewer | Change made |
|  | 213 | km | The change was made in the first revision in the entire manuscript. | The manuscript was re-checked to ensure this correction was affected in all sections. |
|  | 215 | Does this mean that there were two trucks, each operating simultaneously? | The term was introduced to indicate that for certain experiments, data was simultaneously collected for facilitate analysis of two aspects. The term may confuse readers and is therefore deleted. | The point is clarified by pointing out that data was recorded to facilitate the analysis. |
|  | 217-218 | Means what? - Does this mean that there was one panel, smaller than the overall size of the trap, and that the panel was moved around at random to occupy different parts of the trap surface? | We agree with the reviewer | Clarity is given by referring the reader to Fig 1 |
|  | 227-228 | comma respectively. Does a traverse have a temperature and humidity? | We agree with the reviewer | Sentence referring to temperature and humidity has been deleted. |
|  | 229 | DEARY ME! It is said in the table of responses that all instances of “Km” have been changed to “km”, but in fact nothing of the sort has been done. | The change was made in the first revision in the entire manuscript. | The manuscript was re-checked to ensure this correction was affected in all sections. |
| Discussion - It appears that the reviewer used the previous version of the manuscript in reviewing this section. However, certain concerns he raises are still valid and are addressed (Line numbers don’t align with the revised manuscript) | 376 | “vehicle-mounted all-blue, all-black, and blue-black sticky panel traps” is hardly clear | We agree with the reviewer | The sentence has been revised and now reads ‘of all-blue, all-black and blue-black trap panels. |
|  | 378 | Catches of sticky traps depend on a sequence of three things: (i) attraction of flies from a distance, (ii) close range orientation towards the sticky surface, (iii) alighting on the surface in a manner that is likely to ensure permanent retention. Is the intention to say that all of these separate responses are not affected by colour. If so, I fear that the intention is awry, and in any case the idea is contradicted in the next sentence. It makes for very confused reading | We agree with the reviewer with the steps outlined. The idea we are trying to communicate is that only landing response is affected by colour. This is clearly demonstrated by the comparison of the blue and black sections on the blue-black panel. However, this preference to land on black is not apparent when all black and all blue panels are used. Therefore, we point out the need to investigate the lack of colour competition in mobile baits by the inclusion of more colours and also the determination of the possible influence of size. | Changes are made to reflect the effect of colour on the three sequences of behaviours. |
|  | 388 | The sentence starting here is of vague meaning in several ways. For example, if one considers the ability of a device to elicit a landing response it would make most sense to assess what proportion of flies near the bait are encouraged to alight. As matters stand, all that is measured is how many flies alight and get stuck with different treatments. It could well be that certain treatments encourage attraction to the vicinity of the bait and also reduce the strength of the alighting response. Strictly speaking, the results offer no reason to suggest that translocation of panels encourages alighting behaviour | We agree with the reviewer. No data to compare alighting on stationary and mobile panels is given in the text. | The sentence is deleted. |
|  | 390 | Italics for G. m. morsitans. | Already done | No action taken |
|  | 396 | objectives ARE. | We agree with the reviewer | Change made |
|  | 418-419 | known to be; on the panel. | We agree with the reviewer | Change made |
|  | 433 | Split infinitive | We agree with the reviewer | Change made |
|  | 436 | Differences Between | We agree with the reviewer | Change made |
|  | 443 | Delete “among other host seeking stimuli” (stimuli do not seek hosts). | We agree with the reviewer | Deletion made |
|  | 444-445 | Perhaps the differences between the geometry of the habitats of the two species is ….. | We agree with the reviewer | Change made |
|  | 448 | Italics for G. morsitans. | Already done |  |
|  | 451 | . Given the skimpy nature of the present MS, I would have thought it safer to say that more work is recommended to confirm the claim that there is indeed a “drastic” difference in the olfactory responses of the two sub-species. | This point was already included in the previous revision (Line 362) | No action taken |
|  | 466. | No evidence has been adduced to support the claim that” all landing flies are secured”. | We agree with the reviewer. | The sentence is revised and now reads ‘Thirdly, BFR catches are influenced by the hand netting ability of operators (17), whilst catch size of the VST is consistent within the effective range of the sticky film. |
|  | 487 | Delete flies |  | Deletion made |
|  | 503 | more rapid and efficient than the BFR for sampling G. morsitans, so making it … |  | Change made |
| References |  | Numbers 34 and 50 would look better without the excessive capitalization | Already done | No action taken |

Reviewer # 2

| S/N | Reviewer Comment | Response from authors | Action taken by authors |
| --- | --- | --- | --- |
| 1 | The authors have addressed my previous comments. I think the manuscript would benefit from editing for language since it is still verbose and unclear in places. I notice that the first reviewer has made detailed comments on the language and I urge the authors to follow the reviewer's advice. | We agree with the reviewer | Efforts have been made to edit for language |
| 2 | I recommend that the authors re-consider their use of the word 'efficient' and 'efficiency'. In most cases they are reporting that catches increased and this is not necessarily due to improvements in the efficiency of the device. I notice that the first reviewer makes a similar comment in their first review but the authors seem to have ignored this comment. | We agree with the reviewer | The word efficient and efficiency have been removed and replaced with effective and effectiveness. |
| 3 | I am not convinced that the results allow the authors to infer much about the host-oriented behaviour of tsetse. The device is mounted on a large vehicle which itself presents a large visual target but the discussion of tsetse behaviour seems to assume that the responses are to the panels only. I think the main value of the paper is that it highlights the utility of vehicle-mounted sticky screens for sampling tsetse. | As indicated by reviewer #1, catches of sticky traps depend on a sequence of three things: (i) attraction of flies from a distance, (ii) close range orientation towards the sticky surface, (iii) alighting on the surface in a manner that is likely to ensure permanent retention. The large moving vehicle attracts tsetse from a distance and is a constant factor for all treatments. No inferences are made on this aspect. However, once at the vicinity of the moving vehicle, tsetse has to orient towards the sticky and alight on it. All inferences made relate to the latter two actions. We do however concede that further evidence is required to support some of the inferences made. This is highlighted in the text. Where an inference is too controversial, the argument is removed. Refer to the responses to divergent opinions for reviewer #1. | The inference that habitat geometry could affect availability of tsetse of panels has been removed from the manuscript. Only arguments that can reasonable be supported by the data are maintained and emphasis is place on undertaking further research to explain observations. |
| 4 | I am not convinced by the arguments that this method is more cost-effective than using traps. The authors ignore several papers by Shaw et al that show that entomological modelling using traps is much cheaper than the $283/km2 they report. | The reference to Shaw et al (4) indicating the cost of trap for control, $283/km^2^, was removed from the manuscript. This was in view that it refers to the cost of control operations rather than surveys. Leak et al.(5), Bouyer et al. (6) and Krafsur (7) all highlight that the cost of surveys using traps generally restricts their use in large scale surveys, especially were teams have to be deployed for long periods away from their stations. However, since the scope of this study did not include a comparison of relative cost between VST and stationary traps, the argument of cost-effectiveness has been removed and a recommendation for research into this area is made. | All references to cost effectiveness have been removed. |
| 5 | I would like to see the various statements that fly-rounds are 'the' recommended method for sampling G. morsitans changed. I suggest they write that fly-rounds are 'a' method used for sampling G. morsitans. Traps are used widely and I suspect that a systematic review of the literature would show that they are more widely used than fly-rounds. | We agree with the reviewer | The change has been made in the text |

References

1.	Byamungu M, Zacarie T, Pondi AMM, Diabakana M, Mcmullin A, Kro T, et al. Standardising visual control devices for Tsetse : East and Central African Savannah species *Glossina swynnertoni* , *Glossina morsitans centralis* and *Glossina pallidipes*. PLoS Negl Trop Dis. 2018;12(9):e0006831.

2.	Oloo F, Sciarretta A, Mohamed-ahmed MM, Kro T, Mcmullin A, Mihok S, et al. Standardizing Visual Control Devices for Tsetse Flies : East African Species *Glossina fuscipes fuscipes* and *Glossina tachinoides*. PLoS Negl Trop Dis. 2014;8(11):e3334.

3.	Mbewe NJ, Saini RK, Torto B, Irungu J, Yusuf AA, Pirk CWW. Sticky small target : an effective sampling tool for tsetse fly *Glossina fuscipes fuscipes*. Parasites and Vectors. 2018;11:268.

4.	Shaw APM, Torr SJ, Waiswa C, Cecchi G, Wint GRW, Mattioli RC, et al. Estimating the costs of tsetse control options: An example for Uganda. Prev Vet Med [Internet]. 2013;110(3–4):290–303. Available from: http://dx.doi.org/10.1016/j.prevetmed.2012.12.014

5.	Leak SG., Ejigu D, Vreysen MJB. Collection of Entomolological Baseline Data for Tsetse Area-Wide Integrated Pest Management Programmes. Rome: FAO/IAEA; 2008.

6.	Bouyer J, Dicko AH, Cecchi G, Ravel S, Guerrini L, Solano P. Mapping landscape friction to locate isolated tsetse populations that are candidates for elimination. PNAS. 2015;112(47):14575–80.

7.	Krafsur ES. Tsetse flies: Genetics, evolution, and role as vectors. Infect Genet Evol. 2009;9(1):124–41.
